# Self-reported mind wandering reflects executive control and selective attention

**DOI:** 10.3758/s13423-022-02110-3

**Published:** 2022-06-07

**Authors:** Guy E. Hawkins, Matthias Mittner, Birte U. Forstmann, Andrew Heathcote

**Affiliations:** 1 School of Psychological Sciences, University of Newcastle, Newcastle, Australia; 2 Department of Psychology, University of Tromsø, Tromsø, Norway; 3 Integrative Model-Based Cognitive Neuroscience Unit, University of Amsterdam, Amsterdam, The Netherlands

**Keywords:** Mind wandering, Executive control, Selective attention, Sustained attention, Decision-making, Self-report, Cognitive model

## Abstract

Mind wandering is ubiquitous in everyday life and has a pervasive and profound impact on task-related performance. A range of psychological processes have been proposed to underlie these performance-related decrements, including failures of executive control, volatile information processing, and shortcomings in selective attention to critical task-relevant stimuli. Despite progress in the development of such theories, existing descriptive analyses have limited capacity to discriminate between the theories. We propose a cognitive-model based analysis that simultaneously explains self-reported mind wandering and task performance. We quantitatively compare six explanations of poor performance in the presence of mind wandering. The competing theories are distinguished by whether there is an impact on executive control and, if so, how executive control acts on information processing, and whether there is an impact on volatility of information processing. Across two experiments using the sustained attention to response task, we find quantitative evidence that mind wandering is associated with two latent factors. Our strongest conclusion is that executive control is impaired: increased mind wandering is associated with reduced ability to inhibit habitual response tendencies. Our nuanced conclusion is that executive control deficits manifest in reduced ability to selectively attend to the information value of rare but task-critical events.

## Introduction

Mind wandering is ubiquitous in everyday life. Some estimates indicate that it occupies up to 30–50% of our waking hours and pervades almost all daily activities (e.g., Killingsworth & Gilbert 2010). Unlike many other cognitive activities, however, mind wandering can only be manipulated indirectly through conditions that are thought to make it more or less likely. This places the scientific study of mind wandering in a unique and challenging position: its occurrence is unpredictable and fleeting, yet its consequences can be substantial, such as attention lapses during safety critical operations. From lab-based studies, we know that prior to self-reported off-task thoughts relative to on-task thoughts, people tend to have more variable response times (e.g., Bastian & Sackur 2013), greater likelihood of missing target stimuli (e.g., Cheyne, Solman, Carriere & Smilek 2009) and false alarming to non-target stimuli (e.g., McVay & Kane 2012), as well as differential neural activation primarily in the default-mode network (Christoff, Gordon, Smallwood, Smith & Schooler 2009; Mittner et al. 2014; Groot et al. 2021) and the frontoparietal control network (Spreng, Stevens, Chamberlain, Gilmore, & Schacter 2010).

Of the empirically observed performance decrements that accompany mind wandering, the extant literature has emphasized a central role for behavioral variability—in particular, response time (RT) variability—as a robust behavioral marker of mind wandering across different tasks (e.g., Boayue et al. 2021; Cheyne, Solman, Carriere & Smilek 2009; Esterman, Noonan, Rosenberg, & Degutis 2013; Mittner et al. 2014). This behavioral observation is typically assumed to be the outcome of more variable cognitive processing; that is, stimuli are subject to noisier internal evaluation processes, which generates greater behavioral variability from one event to the next. This is, however, a conclusion largely driven through descriptive analyses and verbal theorizing. An early exception, McVay & Kane (2012), used an evidence accumulation model, the Linear Ballistic Accumulator (LBA; Brown & Heathcote 2008), to explore cognitive-process explanations of the finding that off-task thoughts are related to more skewed response time (RT) distributions in the Sustained Attention to Response Task (SART; Hawkins, Mittner, Forstmann, & Heathcote 2019). The SART is a go-nogo decision task with a very low proportion of nogo (target) stimuli. In the LBA, a decision is made when a linearly accumulating evidence total reaches a threshold amount. McVay & Kane (2012) found that trial-to-trial variability in the accumulation rate, which increases RT-distribution variability and skew, was more strongly correlated with off-task thoughts than any other LBA parameter, supporting the variable-cognitive-processing hypothesis.

The SART is of interest because it highlights the executive-control processes necessary to overcome the habit of responding, which is induced by go trials being more common than nogo trials. Throughout, our use of *executive control* refers to the higher-order cognitive process that monitors and intervenes to ensure lower-order response patterns in a goal-directed task remain appropriate. Executive resource theories (Smallwood & Schooler 2006; Teasdale et al. 1995) assume mind wandering redirects a variable amount of the finite pool of executive resources to internally focused cognition, reducing performance and increasing variability in tasks such as the SART that rely on these resources. Executive failure theories (McVay & Kane 2010, 2012; Smallwood 2010) propose that proactive-control processes, which maintain focus on goal-directed thoughts and behaviors necessary for tasks like the SART, can sometimes fail due to mind wandering, causing goal neglect (Duncan, Emslie, Williams, Johnson, & Freer 1996) that again decreases performance and increases variability in responding. Mittner et al. (2014) also used evidence-accumulation modeling to investigate mind wandering in the stop-signal task, which like the SART requires executive control on rarely occurring trials where a signal presented after the choice stimulus requires a choice response to be withheld. Like the LBA, their racing-diffusion model assumes a race-to-threshold among evidence-accumulation processes, but instead of varying trial-to-trial, rates vary from moment-to-moment during accumulation. They found that the occurrence of off-task thoughts, both as measured by self-report and predicted with the aid of physiological measures, were associated with decreased mean rates of accumulation and decreased evidence thresholds.

Cognitive-model based analyses are attractive because they promise to directly identify the association between mind wandering and characteristics of psychological processes, something that can be ambiguous when looking at behavioral data alone. For example, there is a pervasive positive correlation between the mean and variability of RT (Wagenmakers & Brown 2007), and both can be simultaneously affected by different cognitive-model parameters (e.g., increases in both accumulation rate means and variability lead to greater RT mean and variability). However, recent research has revealed problems with applying standard evidence-accumulation models like the LBA and racing diffusion to both the stop-signal task (Matzke, Logan, & Heathcote 2020) and the SART (Hawkins, Mittner, Forstmann, & Heathcote 2019). Here we use the newly proposed Timed Racing Diffusion Model (TRDM), which has been shown to provide a sound description of SART performance in the presence of mind wandering (see Hawkins & Heathcote 2021) to investigate the relationship between processing variability and other aspects of cognitive processing and self-reported mind wandering.

The TRDM proposes that decision-making is driven by three racing diffusion processes: a traditional evidence process consisting of two accumulators that evaluates stimulus identity (i.e., go vs. nogo), and a timing accumulator that tracks the passage of time throughout a decision. A response is withheld if the nogo accumulator wins the race (i.e., a nogo stimulus is identified), and otherwise a go response is made, either because the go accumulator wins or because the timing accumulator wins. The timing accumulator represents the amount of time one is willing to commit to collecting evidence. In the standard TRDM, if that time is exceeded a response is guessed, and given that a go response is most often appropriate in the SART we assume that guesses are biased toward always making a go response.^1^ The timing component is necessary to account for the unusual form of RT distributions that occur in the SART, which cannot be accommodated by traditional evidence-accumulation processes alone (see also Hawkins et al., 2019). The TRDM enables us to examine the relationship between mind wandering and two types of variability in latent processing, in the evidence process or in the timing process. Throughout, we refer to processing *volatility* instead of processing variability to clearly distinguish between the observed data (behavioral variability) and its latent generator (processing variability).^2^

The evidence process of the TRDM allows us to examine the roles of executive control and selective attention. If executive control is important, mind wandering should be more closely related to the rates of accumulation for rarely occurring nogo stimuli than for the more common go stimuli. This is because executive control is reflected in the capacity to successfully inhibit the habitual tendency to respond to frequently occurring non-target (go) stimuli when the rare target (nogo) stimulus appears. Selective attention is reflected in the pairing of particular responses to particular stimuli. For each type of stimulus—go and nogo—there are two evidence rates—one for each type of response: either matching rates (i.e., the nogo accumulator for nogo stimuli and the go accumulator for go stimuli) or mismatching rates (i.e., the go accumulator for nogo stimuli and the nogo accumulator for go stimuli). If mind wandering reduces the ability to bring selective attention to bear to filter out misleading information, mismatching rates will increase. If instead mind wandering reduces the ability of selective attention to focus on relevant information, matching rates will decrease. Hence, we compare the association of mind wandering to go-stimulus vs. nogo-stimulus rates to test the role of executive control, and the association of mind wandering to matching vs. mismatching rates to test the role of selective attention.

The timing process of the TRDM also affords a novel test of the executive control vs. processing volatility accounts. The rate at which time is perceived to pass also reflects executive control, since control is necessary to maintain a well-calibrated sense of time and avoid reverting to habitual actions unless necessitated by a slow choice process. In the SART, one must calibrate the speed of their timer to the expected time required to process task-relevant stimulus information, where a timer that runs too quickly will generate an abundance of premature responses. The timing process of the TRDM also permits a final test of processing volatility—in this instance, the variability with which time is perceived to pass.

To discriminate between the competing theories, we simultaneously analyze two streams of data—SART performance and self-reported mind wandering—as observable outcomes of an integrated latent cognitive process. We propose a cognitive model for the two streams of data and structurally bind the parameters of the two models. This approach is integrative in the sense that data from the behavioral task bear on parameter estimates of the self-report model and self-report data bear on parameter estimates of the TRDM, via a linking function. The ‘best’ parameter estimates are thus those that maximize the joint likelihood of the two streams of data. Such ‘joint modeling’ is increasingly common in the cognitive-neuroscience literature to link behavioral and neural data (Turner, Forstmann, & Steyvers 2019), but its application as a means of linking multiple streams of behavioral data is much less common in the psychology literature (though see Kvam, Romeu, Turner, Vassileva, & Busemeyer 2021; Wall et al. 2021). The primary hypothesis test comes from the structural link between parameters of the TRDM and parameters of the self-report mind wandering model. To this end, we perform model comparison to identify which latent components of processing in the sustained attention task are most strongly associated with self-rated mind wandering during ongoing task performance.

## Method

We develop and evaluate a model that simultaneously generates predictions for sustained attention data (choices, response times) and self-report mind wandering data (Likert scale ratings) in a single, coherent framework. We first describe the design of the experiments we consider followed by details of the cognitive models of each of the two types of data, and of the function that binds them. Data and analysis code are available at https://osf.io/f7vyu.

### Data

We re-evaluated performance in the SART. The SART’s very low proportion of nogo (target) stimuli induces a pattern of rapid, repetitive responding with key properties including very fast responses, unconventionally shaped RT distributions, and error responses that are much faster than correct responses (Hawkins et al., 2019).

We analyzed two previously published SART data sets. We refer to these as Experiments 1 and 2 and present them in parallel as they had similar designs. We summarize the experimental designs here and refer the reader to the primary sources for complete task details; Experiment 1 was first reported in Hawkins et al. (2019) and Experiment 2 was first reported in Boayue et al. (2020). Experiment 2 was conducted in a brain stimulation context and Experiment 1 was conducted in a regular lab-based environment, without stimulation.

Each SART trial displayed a single digit, sampled from the digits 1–9 (Experiment 1) or 0–9 (Experiment 2). Participants were instructed to respond (press a button on the keyboard; go trials) to all digits (i.e., non-targets) *except* to the target digit 3. When the target stimulus was presented, participants were instructed to withhold their response (i.e., do nothing; nogo trials). Go and nogo trials were pseudo-randomly presented subject to the constraint that multiple nogo trials did not occur within a small window. In Experiment 1, 19 participants completed 640 go trials and 80 nogo trials. In Experiment 2, 192 participants completed 1000 go trials and 24 nogo trials. Experiment 2 data had a very small proportion of very slow responses, which were removed from further analysis; specifically, .24% of trials with responses slower than 1.5s, which is exceptionally slow in the SART (cf. *y*-axes in upper row of Fig. 2). No trials were removed from Experiment 1 data.

Interspersed throughout the SART, participants were occasionally presented with ‘thought probes’. In Experiment 1, thought probes asked “Where was your attention during the previous trials?” with responses given on a four-point Likert scale with labels “on task” (position 1) and “off task” (position 4). In Experiment 2, thought probes asked “To what extent have you experienced task-unrelated thoughts prior to the thought probe?” with responses also given on a four-point Likert scale with labels “minimal” (position 1) and “maximal” (position 4). Thought probes were pseudo-randomly presented subject to the constraint that multiple probes did not occur within a small window. Participants completed 20 thought probes in Experiment 1 and 24 thought probes in Experiment 2.

### Modeling approach

#### Self-report model

**Fig. 1:**
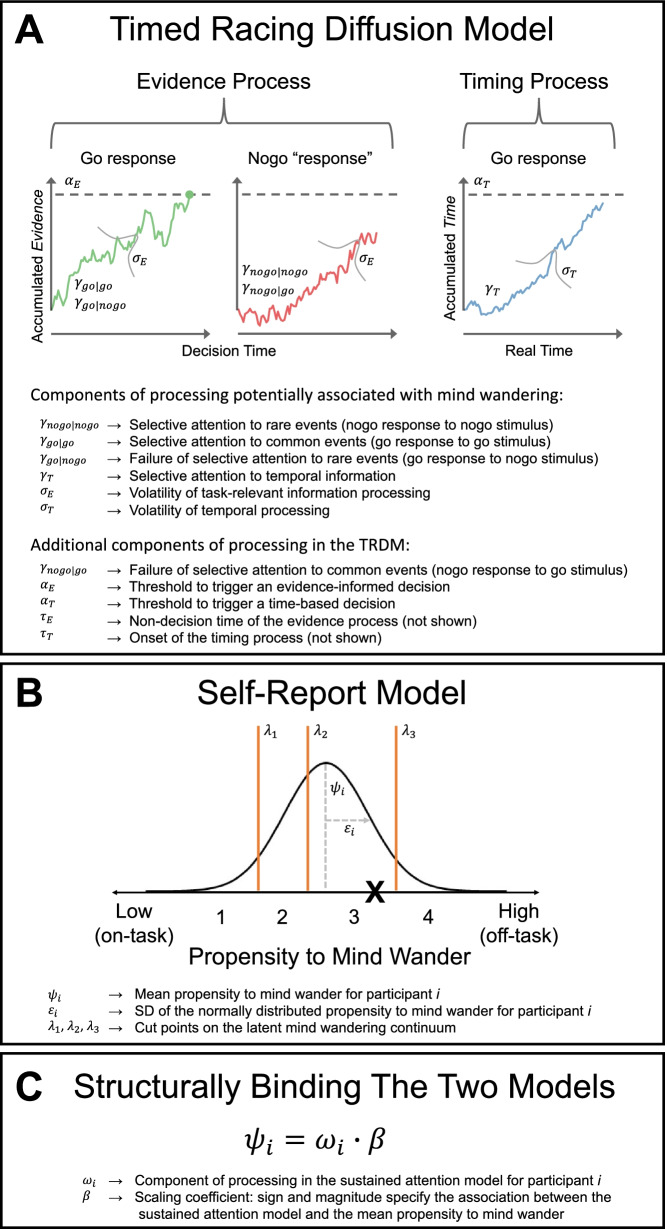
Schematic overview of the joint modeling framework. See text for details. Available at https://tinyurl.com/2p8u987x under CC license https://creativecommons.org/licenses/by/4.0/

We assumed self-report responses to thought probes were generated from a Thurstonian ‘strengths’ model, also known as ordinal probit regression (for similar approach see Boayue et al. 2020). The Thurstonian model assumes the construct of interest is normally distributed along a latent continuum; in our application, the continuum represents the propensity to mind wander—see Fig. 1B. When probed about the focus of attention, the latent continuum is separated with $k-1$ thresholds or ‘cut’ points to divide it into *k* bins for a Likert scale with *k* possible response options. To respond to the thought probe, a sample is taken from the latent distribution and the bin in which the sample falls determines the position selected on the Likert scale. Figure 1B illustrates an exemplar thought probe trial where the random sample from the latent distribution is shown with an **X**, which leads to a response of ‘3’.

#### Timed racing diffusion model

We assumed choices and RTs in the SART were generated from the TRDM—see Fig. 1A. Rates for the go and nogo accumulator are estimated for both go stimuli ($\gamma _{go|go}$ and $\gamma _{nogo|go}$, respectively), and nogo stimuli ($\gamma _{go|nogo}$ and $\gamma _{nogo|nogo}$, respectively). Each evidence accumulator has independent moment-to-moment normally distributed variability with the same standard deviation $\sigma _E$, which we refer to as volatility in processing task-relevant information. Evidence is independently accumulated for the go and nogo choices to an evidence threshold ($\alpha _E$), with the first accumulator to cross threshold dictating the decision (go, nogo) and the time of the decision (for go responses; nogo responses have no observed RT), given the timing process has not already crossed its threshold. The evidence process finishing time is shifted by $\tau _E$ representing the time required for processes outside of evidence accumulation, like encoding the stimulus and motor preparation to generate a response.

The TRDM includes a latent process measuring the passage of time, which acts in a similar manner to the analogous components of processing in the evidence process. The speed at which time is perceived to pass is determined by the timing rate ($\gamma _T$). Timing information accumulates with the same form of moment-to-moment variability as the evidence process, with standard deviation $\sigma _T$, which we refer to as volatility in temporal processing. Accumulation continues until it crosses the timing threshold ($\alpha _T$), which represents the amount of time one is willing to commit to a decision. If the timing process crosses threshold before the go or nogo evidence accumulators, it halts the evidence process and immediately generates a go response. As with the evidence process, the timing process is also shifted by $\tau _T$.

The TRDM as described has 11 parameters, but we imposed a number of constraints to simplify the model. We did not freely estimate the onset time or the threshold of the timing process, instead fixing them to constants ($\tau _T=0$s, $\alpha _T=1$). We also set the mean evidence threshold to a fixed value as the scaling parameter ($\alpha _E=1$). Nogo responses to go stimuli were extremely rare, so we assumed $\gamma _{nogo|go}=0$ with no loss of descriptive adequacy. Together, this reduced the TRDM to seven parameters to estimate from data for each participant.

#### Structurally binding the TRDM and self-report model

We structurally bound the latent propensity to mind wander (Thurstonian model) to latent components of processing in the SART (TRDM)—see Fig. 1C. This allowed us to test hypotheses about the association between individual participant variability in latent components of processing and individual participant variability in self-reported mind wandering. Our approach was to bind one TRDM parameter at a time to the mean of the Thurstonian mind wandering continuum, which generated six independent joint models. Each of the six joint models linked a different TRDM parameter to thought probe responses, so throughout we refer to each joint model by the TRDM parameter linked to probe responding. We did not explore a joint model that bound the non-decision time parameter to the latent mind wandering continuum as we could find no reasonable a priori hypothesis for this association.

Three of the joint models investigated the association between executive control and mind wandering. The first of these bound the propensity to mind wander to the rate for correctly identifying target (nogo) stimuli ($\gamma _{nogo|nogo}$). In this model, we expected participants who had greater executive control were less likely to mind wander and so would overcome the habitual go response and selectively attend to rare target stimuli. In a second model, we bound mind-wandering propensity to the rate for *incorrectly* identifying target (nogo) stimuli as a non-target ($\gamma _{go|nogo}$)—the mismatching rate. We expected that a larger mismatching rate, reflecting an inability to selectively filter out misleading information, would be associated with greater rates of mind wandering. The third model bound the propensity to mind wander to the rate of the timing process ($\gamma _T$). We expected that a larger (more rapid) timing rate reflects a poorly calibrated sense of time, making participants likely to revert to the habitual action of responding. Thus, we expected that greater timing rates would be associated with greater rates of mind wandering. The final model pertaining to TRDM rates was not related to executive control. Rather, it bound mind-wandering propensity to the rate to correctly identify non-target (go) stimuli ($\gamma _{go|go}$), which tests whether mind wandering impacts the ability to selectively attend to commonly occurring task-relevant information. We expected greater selective attention to the frequently occurring non-target stimuli would be associated with reduced mind wandering.

The final two joint models investigated the association between processing volatility and mind wandering. In the fifth model, we bound mind-wandering propensity to volatility in processing task-relevant information ($\sigma _E$). We expected greater volatility in evidence processing would be associated with greater rates of mind wandering. In the sixth and final model, we bound mind wandering propensity to volatility in processing time-based information ($\sigma _T$). We expected greater volatility in temporal processing would be associated with greater rates of mind wandering.

We independently estimated the six joint models and used quantitative model comparison to determine which binding most parsimoniously captured trends in both streams of data, and therefore which hypothesis about the association between a latent component of processing and propensity to mind wander was best supported by the data. In all cases, we simultaneously estimated participant and group-level parameters in a hierarchical Bayesian framework. This approach is critical to our application: individual-participant parameters are essential to explain behavioral performance, yet some parameters of the joint model are only identifiable at the group level (i.e., when constrained to a common value across participants).

We assume presentation of a thought probe on trial *j* prompted participant *i* to sample a value from a latent normal distribution with mean $\psi _i$ and standard deviation $\epsilon _i$, $z_{ij} \sim N(\psi _i, \epsilon _i)$. The participant-specific mean of the normal distribution was determined by the $i^{th}$ participant’s value of the parameter to be linked from the TRDM, which we denote $\omega _{i}$, and a scaling parameter $\beta$ that was estimated at the group level (hence it has no subscript for participant) such that $\psi _i= \omega _{i} \cdot \beta$. We use $\omega$ as generic shorthand to refer to the individual TRDM parameter bound to the thought probe response process in each of the six joint models. That is, $\omega _i=\gamma _{i,nogo|nogo}$ in the model that tests the association between mind wandering and selective attention to rare events, $\omega _i=\gamma _{i,go|go}$ in the model testing the association between mind wandering and selective attention to common events, and so on for the six TRDM parameters (models) described earlier. The mapping from the sampled value $z_{ij}$ to a response on the four-point Likert scale on trial *j*, $p_{ij}$, was determined by the relative position of $z_{ij}$ between three cut points, $\lambda _1$, $\lambda _2$ and $\lambda _3$, which were estimated at the group level (so again with no subscript for participant):

$$\begin{aligned} p_{ij} = \left\{ \begin{array}{llll} 1 &{} \text {if } z_{ij} \le \lambda _1, \\ 2 &{} \text {if } \lambda _1< z_{ij} \le \lambda _2, \\ 3 &{} \text {if } \lambda _2 < z_{ij} \le \lambda _3, \\ 4 &{} \text {if } z_{ij} > \lambda _3. \end{array}\right. \end{aligned}$$

The Appendix describes all estimation details including discussion of parameter identification considerations, specification of prior distributions, and details of the Markov-chain Monte-Carlo (MCMC) sampling algorithm. We quantitatively compared models with the Deviance Information Criterion (DIC; Spiegelhalter, Best, Carlin, & Van Der Linde 2002). DIC accounts for model flexibility both in terms of the number of estimated parameters (parametric complexity) and the way in which those parameters interact (functional-form complexity). The model with the lowest DIC is preferred.

## Results

The joint models were developed using Experiment 1 data and without access to Experiment 2 data. We therefore treat the data from Experiment 2 as a ‘test set’ to evaluate the validity of the model. For this reason, and the much larger sample size of Experiment 2, we place more confidence in the model comparison outcomes of Experiment 2.

### Model comparison

Across both experiments, we found strong evidence that self-reported mind wandering is most strongly associated with the executive control required to overcome habitual actions. Table 1 shows the DIC for the six joint models. In Experiment 1, according to DIC the best explanation of the data was that self-reported mind wandering is associated with reduced capacity to selectively filter out misleading information—a larger mismatching rate for non-target (go) responses to target (nogo) stimuli; higher $\gamma _{go|nogo}$ generates more errors of commission. The second-best explanation of Experiment 1 data assumed that mind wandering is associated with weaker selective attention to rare events—a lower matching rate for target responses to target stimuli; higher $\gamma _{nogo|nogo}$ generates greater rates of target detection and lower rates of errors of commission. The DIC difference between the first- and second-placed models was only eight units, which is generally not considered strong evidence (Pratte, Rouder, & Morey 2010). In contrast, in Experiment 2 there was strong evidence for an association between mind wandering and selective attention to rare events ($\gamma _{nogo|nogo}$), which had a DIC-difference 145 units better than the second most preferred model—selectively filtering misleading information ($\gamma _{go|nogo}$). The top two performing models relate to processing nogo stimuli, so when considered together they provide very clear evidence that executive control is required to overcome habitual response tendencies induced by the frequent non-target (go) stimuli.

The remaining four models provided a much poorer explanation of the data, with DIC differences at least fivefold worse than the top two-ranked models. This includes theories proposed as an explanation of performance in the presence of mind wandering; in particular, an association with the volatility of processing task-relevant information ($\sigma _E$). This speaks against the oft-stated relationship between processing variability and performance in the presence of mind wandering. We also observed weaker support for theories relating mind wandering to selective attention to common events ($\gamma _{go|go}$), and selective attention to and volatility of processing temporal information ($\gamma _T$, $\sigma _T$).

We conclude there is very strong evidence for an association between self-reported mind wandering and the executive control required to inhibit habitual (go) responses to infrequent target stimuli (i.e., strong evidence for the two joint models related to evidence rates for nogo stimuli). There is also evidence that the aspect of executive control most strongly affected is the ability to filter out misleading information ($\gamma _{nogo|nogo}$).

**Table 1:** Model comparison for the six joint models in Experiment 1 and 2

	$\Delta DIC$		Scaling coefficient ($\beta$)	
Hypothesized association with self-reported mind wandering	Expt. 1	Expt. 2	Expt. 1	Expt. 2
Executive control & selective attention to rare events ($\gamma _{nogo|nogo}$)	8	0	−.29 ( −.38, −.21)	−.41 ( −.46, -.37)
Executive control & failure of selective attention to rare events ($\gamma _{go|nogo}$)	0	145	−.65 ( −.81, −.50)	−.62 ( −.69, −.56)
Volatility of task-relevant information processing ($\sigma _E$)	41	712	2.04 (1.57, 2.51)	.59 (.44, .75)
Executive control & selective attention to temporal information ($\gamma _T$)	26	758	−.27 ( −.53, −.04)	−.35 ( −.42, −.26)
Selective attention to common events ($\gamma _{go|go}$)	30	767	−.64 ( −.82, −.48)	−.09 ( −.12, −.06)
Volatility of temporal processing ($\sigma _T$)	33	848	.73 (.39, 1.12)	.60 (.45, .77)

Columns 2 and 3 show DICs zero-referenced to the DIC-preferred model in each experiment such that positive values indicate a poorer explanation of the data. Columns 4 and 5 show the posterior mean and 95% credible interval of the scaling coefficient ($\beta$) in each experiment

### Descriptive adequacy

**Fig. 2:**
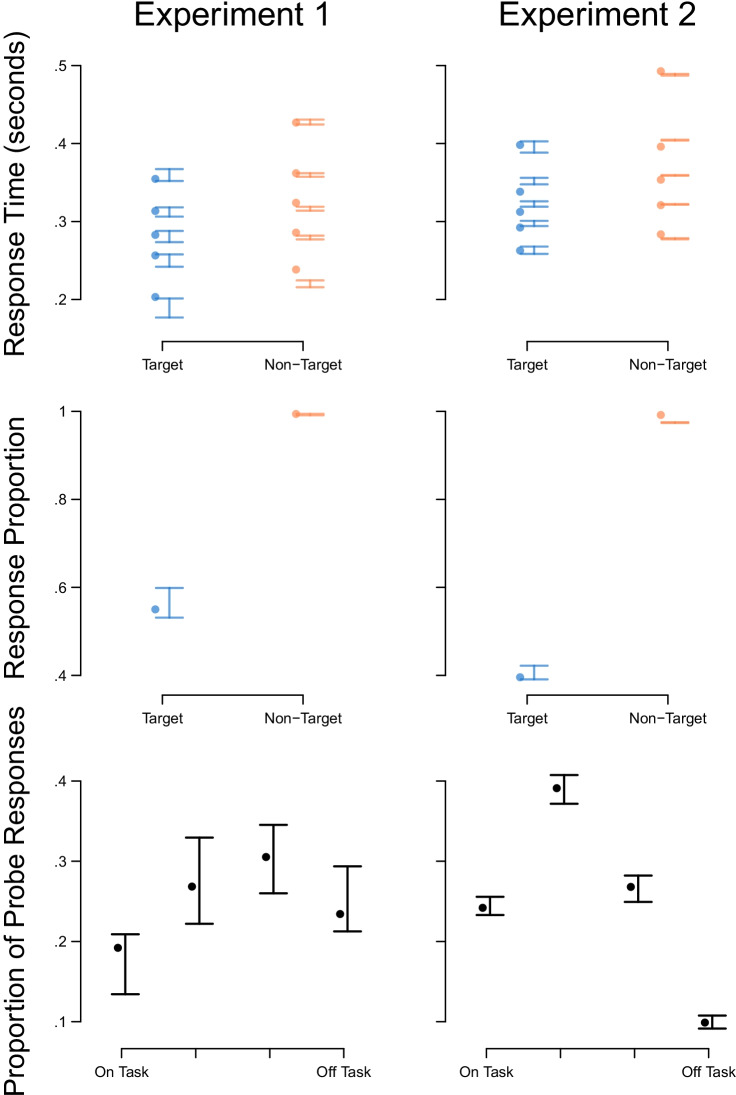
Descriptive adequacy of the DIC-preferred model of self-reported mind wandering associated with executive control and selective attention to rare events ($\gamma _{nogo|nogo}$) for Experiments 1 and 2. Observed data are shown with dots and 95% credible intervals of the posterior predictive distribution are shown with lines and bars. The upper row shows response times where the *y*-axes show the $10^{th}$, $30^{th}$, $50^{th}$ (i.e., median), $70^{th}$ and $90^{th}$ percentiles of the distribution for target (incorrect; blue) and non-target (correct; orange) responses. The middle row shows the proportion of observed responses for targets and non-target stimuli. The lower row shows thought probe responses where the *x*-axes show the four response options of the thought probe response scales and the *y*-axes show the proportion of times each probe response was given, on average. Available at https://tinyurl.com/chy47xr8 under CC license https://creativecommons.org/licenses/by/4.0/

**Fig. 3:**
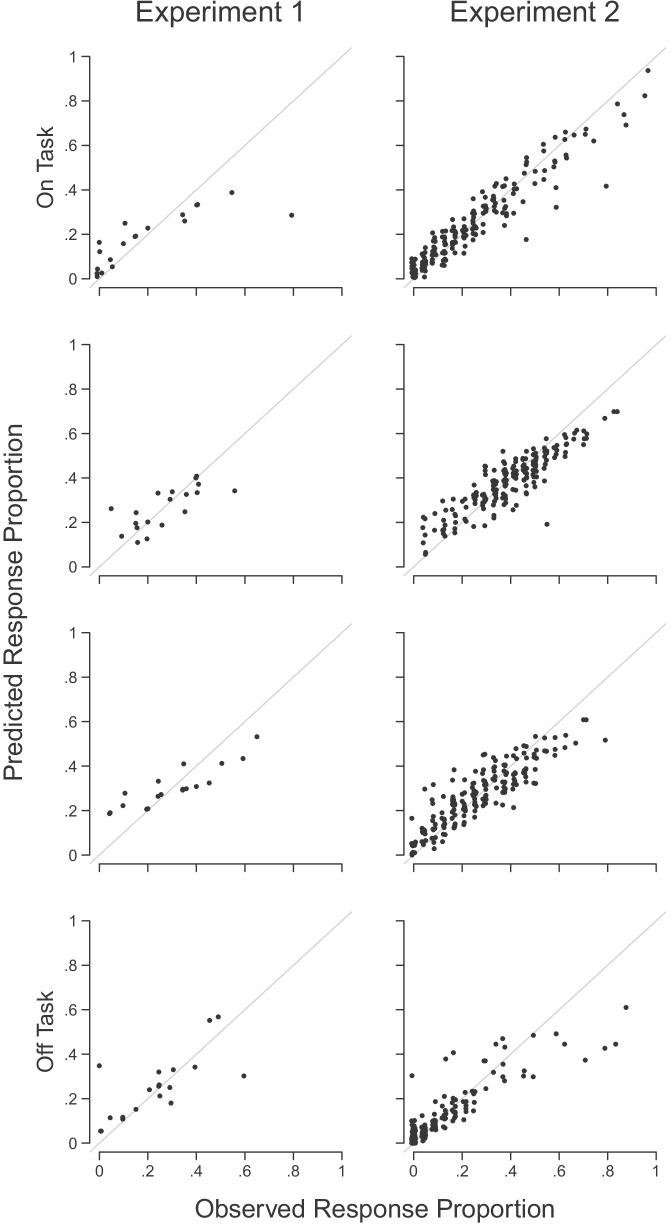
Descriptive adequacy of the DIC-preferred model for the self-report data of individual participants in Experiments 1 and 2. Rows represent the four response options of the thought probe scale (*on task* in the uppermost row through to *off task* in the lowermost row) and columns show the two experiments. Dots in each panel show the observed proportion of times each participant gave each probe response (*x*-axes) against the mean of their posterior predictive distribution (*y*-axes). Perfect model predictions fall on the identity lines. Available at https://tinyurl.com/2muhstuc under CC license https://creativecommons.org/licenses/by/4.0/

Figure 2 shows that an explanation of self-reported mind wandering based on executive control and selective attention to rare events captured all key qualitative and almost all quantitative trends in RT, accuracy, and self-report thought probe data. That is, the RT, choice proportion and thought probe data (dots) fall within the uncertainty region of the posterior predictive distribution (bars) for almost all summary statistics. RT distributions for the group (upper row) were calculated via quantile averaging the individual participant observed and posterior predictive data. The model captured the key trends in the location and shape of the distribution. As expected, the proportion of observed responses for targets, which were errors of commission, was much lower than the proportion of observed responses for non-targets, which were correct identifications—a trend that the model replicated. Self-reported task-unrelated thoughts demonstrated a ‘bow’ effect in both experiments: relatively few reports of completely on- or off-task thoughts, with a peak at an intermediate level of task-related thoughts. The model very closely captured the quantitative effects in the self-report data at the group level, shown in Fig. 2, and for individual participants, shown in Fig. 3. This is important to demonstrate since this is the novel addition to the quantitative modeling approach in this paper. Taken together, these results indicate the DIC-preferred model captured the key trends in the RT, choice, and thought probe data in both experiments.

### Psychological interpretation

There were large individual differences in the proportion of commission errors—observed responses to target stimuli. For instance, in Experiment 1 the mean proportion of commission errors was .55 with a range across participants of .20 to .95. The TRDM captures these between-person differences primarily through selective attention to rare events such that greater nogo rates to nogo stimuli generates fewer commission errors. In support of this, the DIC-preferred model had a reliable negative correlation between the proportion of commission errors observed in data and individual participant estimates of selective attention to rare events: Experiment 1 $r_{s}=-.829$, 95% credible interval (CI) [$-.910$, $-.718$] and Experiment 2 $r_{s}=-.368$, 95% CI [$-.408$, $-.328$].

In the DIC-preferred model, the component of processing related to selective attention to rare events underlying much of the individual differences also scales the mean of the latent Thurstonian continuum. The posterior mean of the scaling parameter in Experiment 1 was $\beta = -.286$, 95% CI [$-.379$, $-.200$] and in Experiment 2 was $\beta = -.412$, 95% CI [$-.466$, $-.373$]. This indicates that executive control, and in particular selective attention to rare events, are also strongly associated with the probability of self-reporting task-unrelated thoughts. Indeed, there was a strong negative correlation between individual participant mean thought probe responses and individual participant estimates of selective attention to rare events: Experiment 1 $r_{s}=-.809$, 95% CI [$-.906$, $-.689$] and Experiment 2 $r_{s}=-.912$, 95% CI [$-.929$, $-.893$].

## Discussion

We have shown that a joint quantitative model of sustained attention and self-report data provides a fine-grained tool to investigate and test hypotheses about latent cognitive factors associated with mind wandering during ongoing task-focused performance. We successfully explained self-reported task-unrelated thoughts even though the joint model did not include an entirely separate cognitive mechanism to quantitatively explain thought probe responses. Rather, the joint model leveraged the psychological mechanisms encapsulated in a cognitive process model of decision-making (TRDM) by structurally ‘adding on’ a response mechanism for thought probe responses (Thurstonian response model). This finding suggests that the latent components of processing in a cognitive model of decision-making contain information about the extent to which a participant is attending to their ongoing task. This association has often been stated in qualitative terms. However, to our knowledge, this is the first direct test of a quantitative link between self-reported task-related attention and components of processing in a cognitive model of decision-making. While our modeling approach inherits the downfalls of any correlational analysis, our capacity to identify which latent cognitive process among a set of candidate latent processes is most likely impaired by mind wandering considerably reduces the space of possible ‘unknown third variables’ that may mediate previously observed empirical associations with mind wandering.

Our strongest result is that the propensity to mind wander during ongoing performance is negatively associated with executive control in the SART. This conclusion is consistent with the top-two performing models. These models have a primary role for the decision-maker’s ability to inhibit the habitual tendency to respond that is induced by the frequent non-target (go) stimuli. We also found support for a nuanced result about how executive control acts: when people mind wander, we see impairments in the ability to selectively attend to the information value of critical rare events (i.e., targets). In contrast, we found weaker evidence for a theory that associates mind wandering with volatility in processing task-relevant information, in both experiments. Our findings suggest that the previously reported empirical results of increased behavioral variability during episodes of mind wandering in the SART may stem from a less effective executive system rather than volatile processing. Finally, we observed weak evidence for an association between self-reported mind wandering and selective attention to common events, or with selective attention to and volatility in processing temporal information (cf. Table 1).

Key to interpreting our modeling outcomes is understanding that any reasonable psychological process model is unlikely to have one-to-one mappings between latent psychological constructs and observed behavior. In this light, we must acknowledge that changes in manifest variability do not necessarily arise from changes in psychological process parameters that only affect latent variability (volatility). This is because interactions between the latent psychological processes mean that some parameters can simultaneously impact manifest central tendency and variability. In a similar vein, model parameters that affect response withholding can also impact go responding because of interactions in the psychological processes (i.e., winning a ‘race’ between go and nogo responses).

Our results highlight the strength of quantitative methods in elucidating competing psychological theories. We believe that our quantitative framework for jointly modeling multiple streams of data—here, sustained attention performance and self-reported mind wandering—is a step forward for teasing apart the predictions of different theories about the impact of mind wandering (see also Hawkins, Mittner, Boekel, Heathcote, & Forstmann 2015; Hawkins, Mittner, Forstmann & Heathcote 2017).

### Limitations

A limitation of our Thurstonian response model is the assumption of a latent mind wandering continuum with a normally distributed latent state. This assumption is consistent with some theories of mind wandering, such as executive resource theories (e.g., Smallwood & Schooler 2006), though it may be inconsistent with others. For instance, the perceptual decoupling theory proposes that goal-directed activity takes place in one of two states: perceptual coupling where attentional resources are directed to task-relevant sensory inputs, and perceptual decoupling where attentional resources are diverted from sensory inputs toward internally-focused cognition (e.g., Smallwood & Schooler 2015). While there is evidence the latent state may be continuous or categorical (e.g., Zanesco, Denkova, Witkin, & Jha 2020), participants may more reliably report their internal state using categorical reports compared to ordinal responses, such as Likert scales (e.g., Kane, Smeekens, Meier, Welhaf, & Phillips 2021). Nevertheless, even if the latent state is discrete, its extent across time and noise in self reporting may render our continuous latent representation a reasonably good approximation. Furthermore, ordinal response models have provided an excellent description of data reported on ordinal scales, as found here (Figs. 2 and 3) and elsewhere (e.g., Boayue et al. 2020). We also note that alternative forms of the latent mind wandering state may be identifiable given appropriate data (e.g., Hawkins, Mittner, Forstmann & Heathcote 2017), and a fruitful direction for future research may be to directly compare such alternative latent forms.

A second limitation of our modeling approach is the lack of temporal dependence in the latent representation of the mind wandering state. Empirical results suggest that self-reported mind wandering shows such temporal dependence throughout the course of an experimental session (e.g., Boayue et al. 2020; Welhaf et al. 2020; Zanesco et al. 2020; Zanesco 2020). The most straightforward way to address this problem may be to incorporate statistical time series models, such as autoregression or hidden Markov models, into cognitive process models, such as the TRDM (e.g., Gunawan, Hawkins, Kohn, Tran, & Brown in press; Kucharskỳ, Tran, Veldkamp, Raijmakers, & Visser 2021). However, such an approach may not necessarily be the most fruitful to pursue because it would not provide a cognitive explanation for changes in the frequency and depth of mind wandering with time on task; this would require a deeper psychological theory about temporal effects. We note that although we did not incorporate temporal dependence into our approach, the model still provided an excellent quantitative description of the data.

### Implications and conclusions

A particular strength of our approach is addressing the inability to directly manipulate mind wandering in an experimental context. Mind wandering is typically assessed as a dependent variable in the context of a primary task with thought probes interspersed throughout. Despite measurement as a dependent variable, it has become standard in the mind-wandering literature to treat thought probes as an independent variable with performance in the primary task analyzed as a function of thought probe responses (e.g., Smallwood & Schooler 2006), although there is a more recent trend to treat thought-probe responses as outcome variables (e.g., Boayue et al. 2021; Filmer, Griffin, & Dux 2019). With the standard analytic approach, we have learned a great deal about mind wandering, including its association with greater response variability and errors of commission (Cheyne et al., 2009). Nevertheless, an analytic approach that switches the role of dependent and independent variables has at least two shortcomings. First, it violates the assumptions of many conventional statistical analyses such as ANOVA, which are commonly used to analyze mind wandering-related data, because the data within each cell are not randomly distributed; the dependent variables from the primary task are conditioned on whether they preceded self-reported on- or off-task thoughts. This approach also tends to produce unbalanced cell sizes because self-reported mind wandering is rarely uniformly distributed along the thought probe response scale (e.g., Hawkins, Mittner, Forstmann, & Heathcote 2019).

Second, the standard analytic approach is theoretically unsatisfying because it cannot explain *how* or *why* people generate their mind wandering self-reports. The cognitive psychology literature as a whole is replete with quantitative process models that explain behavior in different cognitive domains. Yet mind wandering—another cognitive activity—has a distinct lack of proposed cognitive models. Some work has proposed models of mind wandering, most notably within the ACT-R framework (e.g., Taatgen et al. 2021; van Vugt, van der Velde, & ESM-MERGE Investigators 2018). These models are a promising step forward for the literature as they simultaneously generate predictions for sustained attention and self-report, which will lead to more integrated, unified explanations of cognition. However, to date, these existing approaches have tended to explain performance at the level of group averages rather than individuals, and they prioritize explanation of self-reported mind wandering at the expense of a descriptively adequate explanation of sustained attention. To our knowledge, ours is the first integrated framework that generates descriptively adequate and quantitatively precise predictions for performance in a primary cognitive task *and* self-reported mind wandering. This specification enabled us to test precisely-defined theories about the association between self-reported mind wandering and task performance in the SART, finding a primary role for executive control and selective attention to rare events.

Our findings lend support to a ‘dynamic balance’ theory of mind wandering (cf. Mittner, Hawkins, Boekel, & Forstmann 2016). At each moment in time, people estimate the utility of current actions weighed against their expected reward, and consider: what’s in it for me to engage with the task relative to expending cognitive effort elsewhere? Staying on task for long periods is costly (effortful, boring, etc.) and if maintaining task-related focus does not lead to positive outcomes (tangible rewards, satisfying curiosity, novelty value, etc.) then off-task thoughts and mind wandering ought to become more attractive over time. We believe this hypothesized utility balancing process is a ‘meta-process’ in that it drives attentional *and* mind wandering processes. In this sense, our proposal does not claim that mind wandering causes failures of executive function or that failures of executive function cause mind wandering. Rather, the utility-monitoring meta-process will upregulate one process (attentional or mind wandering) and downregulate the other as a function of the momentary expected reward. Similar utility-monitoring processes have been proposed in previous cognitive models of performance under distraction (e.g., Gunzelmann, Gross, Gluck, & Dinges 2009). While our quantitative approach does not explicitly incorporate such a meta-level balancing process, the work we presented here could be expanded to do so in future research (cf. Hawkins et al., 2017).

Finally, we propose that future research investigates whether the same latent cause is associated with mind-wandering-related performance decrements in other experimental paradigms and contexts—beyond the widely studied SART. In particular, we suspect additional study is needed to determine the extent to which our findings generalize to contexts in which executive control may not be critical to ongoing task performance.

## Data Availability

Data and analysis code for this manuscript are available at https://osf.io/f7vyu. No new experimental data were collected for this manuscript; therefore no experiments were pre-registered.

## Appendix

This Appendix provides additional specification of the joint models described in the main text and complete details of the hierarchical Bayesian parameter estimation scheme.

Compared to a TRDM for the sustained attention data that had seven parameters for each participant, a joint model of the sustained attention and self-report data added one participant-specific parameter ($\epsilon _i$) and four group parameters that were freely estimated from data but constrained to a common value across all participants ($\beta$, $\lambda _1$, $\lambda _2$ and $\lambda _3$). This setup ensured that the model was sufficiently constrained to be identifiable, which is not the case if $\lambda _1$, $\lambda _2$ and $\lambda _3$ were treated as participant-specific parameters. As described in the main text, we specified and independently estimated six joint models where each model corresponded to a different TRDM parameter bound to the mean of the Thurstonian normal distribution for thought probe responses (i.e., $\omega$ cycled through $\gamma _{go|go}$, $\gamma _{go|nogo}$, $\gamma _{nogo|nogo}$, $\sigma _E$, $\gamma _T$, $\sigma _T$).

We estimated model parameters in a hierarchical Bayesian framework using differential evolution Markov chain Monte Carlo (DE-MCMC) sampling with the default settings unless noted otherwise (Turner, Sederberg, Brown, & Steyvers 2013). We took 10,000 posterior samples from each of 30 MCMC chains, with a burn-in period of 5000 samples. During the first 2000 samples of the burn-in period we conducted a “migration” update step for participant-level parameters with probability .05, which helps to remove chains stuck in low likelihood regions of the parameter space (for details, see Turner et al., 2013). We thinned to every $5^{th}$ iteration for a total of 30,000 samples from the posterior distribution of the parameters. Convergence was monitored through visual inspection and the multivariate potential scale reduction factor ($\widehat{R}$; Brooks & Gelman 1998).

For the TRDM, the seven individual-level parameters were log transformed to the real line and sampled hierarchically from Normally-distributed hyper distributions. The prior distributions for the hyper means (superscript $\mu$) and standard deviations (superscript $\sigma$) were

$$\begin{aligned} \gamma ^{\mu }_{go|go},\ \gamma ^{\mu }_{go|nogo},\ \gamma ^{\mu }_{nogo|nogo},\ \gamma _T^{\mu }\sim & {} N(1,\ 1) \\ \tau _E^{\mu },\ \epsilon ^{\mu }\sim & {} N(0,1) \\ \sigma _E^{\mu },\ \sigma _T^{\mu }\sim & {} N(-.5,1) \\ \gamma ^{\sigma }_{go|go},\ \gamma ^{\sigma }_{go|nogo},\ \gamma ^{\sigma }_{nogo|nogo},\ \gamma _T^{\sigma },\ \tau _E^{\sigma },\ \sigma _E^{\sigma },\ \sigma _T^{\sigma },\ \epsilon ^{\sigma }\sim & {} \Gamma (1,\ 1) \end{aligned}$$

where *N*(*a*, *b*) denotes the normal distribution with mean *a* and standard deviation *b* and $\Gamma (c,d)$ denotes the Gamma distribution with shape *c* and scale *d*.

For the Thurstonian model, we estimated four parameters, which were held to the same value across all participants. For each of the six joint models, the prior distribution for the scaling parameter was $\beta \sim N(0,\ 1)$. We freely estimated the lowest cut point on the latent Thurstonian scale as $\lambda _1 \sim N(0,\ 1)$. To impose monotonicity of the cut points we estimated the *difference* between each successive threshold: $\log (\lambda _2 - \lambda _1) \sim N(0,\ 1)$ and $\log (\lambda _3 - \lambda _2) \sim N(0,\ 1)$.

## Footnotes

1 Hawkins & Heathcote (2021) examined both biased and unbiased guessing schemes and found only small differences. In the present case, if a response was withheld with the same (low) probability as a nogo stimulus occurring we would expect little difference in our results.

2 Hawkins & Heathcote (2021) showed strong mimicry in the effects of moment-to-moment and trial-to-trial evidence rate variability and similar performance between diffusion-based and LBA-based versions of their timing model. Hence, the volatility hypothesis that we test could correspond to either moment-to-moment effects, trial-to-trial effects, or some combination of the two.
